# Population-Based Screening for Coeliac Disease in Lithuanian Children from 2009 to 2014

**DOI:** 10.3390/medicina59091630

**Published:** 2023-09-08

**Authors:** Vaidotas Urbonas, Jolita Sadauskaite, Dominykas Varnas

**Affiliations:** Clinic of Children’s Diseases, Faculty of Medicine, Vilnius University, 03101 Vilnius, Lithuania; vaidotas.urbonas@mf.vu.lt (V.U.); jolitasadauskaite@gmail.com (J.S.)

**Keywords:** coeliac disease, prevalence, Lithuania, children

## Abstract

*Background and Objectives.* Coeliac disease is an autoimmune disorder provoked by a dietary group of proteins called gluten in genetically predisposed individuals. Over the past several decades, the prevalence of coeliac disease has been steadily growing and it is now recognized to be occurring worldwide. The prevalence varies greatly between ethnic, racial groups and regionally. Such variability makes local epidemiological studies important for spreading awareness and setting a threshold for suspicion of coeliac disease. We explored the potential application of a quick point-of-care test for the purpose of detecting a presence of IgA class TG2 antibodies for coeliac disease and screening in a Lithuanian pediatric population. Previously, there were no data regarding coeliac disease prevalence in Lithuania. *Materials and Methods.* Overall, we included 1458 children 11–13 years of age from several Lithuanian schools selected randomly in this study. Utilizing one point-of-care test using a single blood sample taken from a fingertip, we identified the existence of IgA class TG2 antibodies. Only children whose parents gave consent were enrolled in the study. Those with positive IgA class TG2-ab were directed to a tertiary hospital for additional clinical assessment and confirmation of suspected coeliac disease. *Results.* A total of two (0.14%) of the 1458 enrolled children were detected with the presence of TG2 antibodies and the coeliac disease diagnosis was further confirmed with histological examination of duodenal biopsy samples. Additionally, we checked that patients had not previously reported any clinical symptoms and signs that could suggest coeliac disease or any other disease of the gastrointestinal tract. *Conclusions.* The detected prevalence of coeliac disease in the Lithuanian pediatric population is 1:729. The rapid finger prick test for the presence of IgA class TG2 antibodies is a reasonable and accurate method to screen for celiac disease in children.

## 1. Introduction

Coeliac disease is an autoimmune disorder that primarily affects the small bowel. It is a result of an aberrant immunological response to dietary gluten in individuals with a genetic susceptibility [1]. Gluten is a general term for a group of proteins found in wheat, barley, and rye, which are not fully digested in human intestines. These partial digestion products—glutenin and gliadin peptides—are immunogenic and can trigger complex immune responses that lead to enteropathy and extraintestinal manifestations [2]. According to the European Society of Paediatric Gastroenterology Hepatology and Nutrition (ESPGHAN) guidelines, diagnostics are based on a positive serological test of IgA class anti-transglutaminase and anti-endomysial (EMA) antibodies and in some cases duodenal biopsy specimen examination is required [3]. Currently, the only treatment is a life-long diet without any gluten.

Historically, it was thought that coeliac disease predominantly occurred in northern and western Europe, but with the betterment of diagnostic methods and greater knowledge regarding the disease it is now recognized to be occurring worldwide [4]. Prevalence depends on the diagnostic methods used and is considered to be 1.4% detected with serological testing and 0.7% after performing duodenal biopsy specimen examination worldwide. In the general population, it varies depending on the geographical location (being highest in Europe and Oceania regions (0.8%), while the smallest is reported to be in South America (0.4%) [5]. Children are affected more frequently than adults (0.9% vs. 0.5%) [6]. Moreover, the incidence has been increasing over the previous decades, and such a rise in numbers of coeliac disease cannot be attributed only to better diagnostic methods and awareness. It may be that changes in gluten consumption and feeding practices are contributing to the increase in coeliac disease prevalence [2,7].

The prevalence of coeliac disease can vary not only between ethnic or racial groups [8], but there are also great regional differences, which highlights the environmental factor significance in the pathogenesis of coeliac disease [9,10]. Such variability in prevalence rates shows the importance of regional epidemiological studies. Data derived from such studies would help to set a threshold for screening and increase awareness. We provide the first data about coeliac disease prevalence in Lithuania. 

## 2. Materials and Methods

### 2.1. Study Methodology and Participant Description

The study was conducted in Vilnius University Children’s Hospital. We screened 1458 schoolchildren (714 girls and 744 boys) 11–13 years old for coeliac disease in two Lithuanian cities, from January 2009 to March 2010 in Vilnius and from September to December 2014 in Alytus. Six separate schools were randomly selected in Vilnius and one in Alytus. We asked 2261 schoolchildren to participate in the study: 1583 from Vilnius and 678 from Alytus. Of these possible participants, a total of 1494 (66.1%) children and 1510 (66.8%) parents signed informed consent forms in order to participate. According to our national requirements, the children were included when both personal and parents’ agreements were received. At the time of the investigation thirty-six students who could potentially have been included in the study were absent from school. The final cohort was made up of 1000 adolescents (496 females and 504 males) from Vilnius and 458 (218 females and 240 males) from Alytus.

The voluntary testing was performed at schools by the same, fifth-year student of medicine from the Vilnius University Faculty of Medicine who was sufficiently informed and trained for this task beforehand. 

The Lithuanian Bioethics Committee (LBC) approved the study recruitment and study protocol. Adherence of all investigatory methods that involve human subjects to the ethical standards of the LBC and the 1964 Helsinki Declaration, along with its subsequent amendments, was maintained.

### 2.2. Coeliac Disease Testing

The testing for the presence of coeliac disease antibodies and total IgA concentration was performed, in line with the guidelines provided by the manufacturer, through the utilization of a commercially available point-of-care test (Biocard™ Coeliac Test, Ani Biotech, Vantaa, Finland). The Biocard test is a qualitative immunochromatographic assay capable of concurrently detecting the presence of coeliac-disease-specific IgA TG2 antibodies and IgA. During the investigation, 10 µL of blood, using a capillary tube, was taken from the study subject’s finger. Then, it was put in a testing tube where capillary blood was mixed with the buffer solution using shaking. The researcher continued by dispensing three drops of the prepared mixture, extracted from the test tube, into the cavity of a single-use chromatographic plate. The results of the test were evaluated shortly after 2 to 5 min. In a previous study, it was shown that the Biocard point-of-care test exhibits a sensitivity of 78.1% and a specificity of 100% in cases of histologically confirmed coeliac disease [11]. Additionally, a sensitivity of 96.7% and a specificity of 93.5% were achieved using preserved patient samples, when compared to the results of EMA antibody and TG2 antibody tests [12]. The more detailed procedure and our results regarding IgA deficiency were published in a separate study and will not be elucidated here [13].

The parents were informed if the presence of IgA class TG2-ab or IgA deficiency was detected. Subsequently, the study subjects were directed to Vilnius University Children’s Hospital (tertiary-level hospital) for additional testing and further clinical investigation (namely upper gastrointestinal endoscopy with biopsies). Positive coeliac disease histology was considered if mucosal changes were classified as Marsh 2 or 3.

During the timeframe of study conduction, a total of 112,990 children between the ages of 11 and 13 years old lived in Lithuania. In total, 15,138 of those children lived in the city of Vilnius [14]. Our objective was to determine the prevalence of coeliac disease with a calculated probability of 90%. To accomplish this task, 1061 children in Lithuania and 1001 in Vilnius had to be included in the study. The projected rate of participation stood at 60%, and for this study, a total of six schools were chosen. We used the program Epi Info statistics (Centers for Disease Control and Prevention, Atlanta, GA, USA) and Statistical Package for the Social Sciences software version 22 (SPSS Inc., Chicago, IL, USA) to analyze the study data. We calculated the celiac disease prevalence rate by placing the total number of cases in the numerator and the entire population count in the denominator. Finally, we determined the 95% confidence intervals for the calculated prevalence rate.

## 3. Results

In total, 1385 (95%) of the participating children belonged to the Lithuanian ethnicity. Both the examiners and the children found the testing procedure to be uncomplicated, and no negative effects were reported with the exception of mild fingertip discomfort due to skin puncture to draw capillary blood. Table 1 displays the distribution of the study group and the occurrence of IgA class TG2 antibodies based on age and gender. There were no notable variations in the number of children or gender distribution among the three age groups.

In total, 2 out of the 1458 children participating in the study (0.14%) had positive test results for the TG2 antibody presence: one boy and one girl (both did not have concomitant IgA deficiency). Subjects were referred to the tertiary hospital and subsequent histological examination of duodenal biopsy specimens revealed coeliac disease in both (Marsh 2 and 3). The asymptomatic girl had been previously diagnosed with coeliac disease but was not compliant with recommended treatment and did not adhere to a strict gluten-free diet. The boy also had no symptoms. Both of these study participants were subsequently placed on a life-long gluten-free diet.

Based on the Biocard test and specific investigations, selective IgA deficiency was diagnosed in four asymptomatic children—a boy and a girl both 12 years old and two boys 13 years old. All of them had normal aTG2 IgG levels. In line with our clinical practice during that period, Esophagogastroduodenoscopy with multiple mucosal biopsies from the small bowel was offered. The Esophagogastroduodenoscopy was performed on two study participants. Each of them had non-pathological microscopic and macroscopic findings. For the remaining two IgA-deficient subjects, examinations were not performed because parents refused endoscopy.

## 4. Discussion

First of all, the primary finding in this investigation was the first data on epidemiology of coeliac disease in Lithuania. The prevalence of coeliac disease in Lithuanian children was 1:729 (0.14%). We chose a cohort of children aged 11–13 years old because most patients develop coeliac disease before the age of 10 [15]. In addition, children of this age are less likely to be afraid of blood sample collection, thus resulting in less stress and a greater parent satisfaction and participation rate. Children from this age group are also either pre-pubertal or at the beginning of their puberty. Celiac disease has a potential to impair pubertal development and growth [5] so the authors believed this age group to be an optimal study group. 

Secondly, the quick fingertip point-of-care test presents a convenient, feasible, and precise approach for screening coeliac disease in a diverse pediatric population. Easily implemented, cost-effective methods for coeliac disease diagnostics could play an important part in mass-screening and gathering epidemiological data [16,17].

A systematic review of the global prevalence of coeliac disease found that the biopsy-diagnosed pediatric coeliac disease prevalence is 0.9%, while the seroprevalence rate is usually higher and is believed to be an overestimation [6]. Studies that were included in the metanalysis showed the prevalence of biopsy-proved coeliac disease ranging from 0.2% to 3.03% in pediatric populations [9,18]. We found a significantly lower prevalence. However, if compared with neighboring countries, the Lithuanian prevalence is only slightly lower: 0.25% in Polish and 0.34% in Estonian children [19,20].

Bearing in mind that many autoimmune conditions have a positive correlation with having another autoimmune condition [5], we decided to further explore these prevalence rates. We reviewed other known autoimmune disease rates and compared them regionally.

In Lithuania, the inflammatory bowel disease (IBD) incidence rate is 8.5/100,000 [21], which is one of the lowest in Europe [22]. Both IBD and coeliac disease are of an autoimmune nature and more prevalent in developed countries. There is also a clear bi-directional correlation between these diseases [23] and evidence is starting to emerge about the causal link of these two conditions [24]. Having this in mind, it seems that our findings regarding coeliac disease prevalence correlate with known epidemiological data about inflammatory bowel disease in Lithuania.

Another autoimmune disorder is type 1 diabetes mellitus (T1DM), which has a positive correlation and proven link to coeliac disease [25]. The prevalence of T1DM in the adolescent and young adult Lithuanian population is 83.5/88.4 (male/female) per 100,000 [26], with the incidence being 10.5 per 100,000 inhabitants per year [27]. It is comparable to neighboring countries with Belarus having 7.26/100,000 per year [28], Poland having 11.9 [29], and Latvia having 7.4 [30]. However, other countries in the region seem to have higher T1DM incidence rates: Estonia—17.2/100,000 per year [31], Finland—42.9 [32], and Sweden—43.9 [33]. The pooled incidence of type 1 diabetes in Europe is 15 per a population of 100,000 [34]. Such data correlates with coeliac disease distribution, Lithuania being on the lower end of the incidence spectrum.

It is worth keeping in mind that data for this study were collected in 2009–2014. Considering the gradually increasing incidence and prevalence of coeliac disease, it may be that current rates are higher in Lithuania and additional epidemiological studies would be needed to confirm that [7]. However, it is the first epidemiological study regarding pediatric celiac disease prevalence in Lithuania and these data can be important for research, awareness, and regional health-related policy making. The most important strength of this study is a nationally representative and well-defined study cohort. A major limitation is the low number of diagnosed coeliac disease cases. This could reduce the study’s statistical accuracy. Another limitation is that two of four children with diagnosed IgA deficiency did not agree to further endoscopic evaluation. Children with IgA deficiency more commonly have concomitant celiac disease and there is a possibility that one or both might have had this comorbidity. In such a case, the prevalence would be 0.21% or 0.27%, respectively, which is lower than in other countries. Finally, even though it is easy to utilize and cost-effective, the sensitivity and specificity of the used Biocard point-of-care test are lower compared to the serum anti-TG2 antibody and anti-endomysial antibody tests. This reduces the impact of this study.

## 5. Conclusions

The prevalence of coeliac disease in the pediatric population of Lithuania is 0.14%. The quick fingertip point-of-care test is suitable and easily appliable for population screening for coeliac disease and is also an accurate and practical method in outpatient settings.

## Figures and Tables

**Table 1:** Arrangement of study participants based on gender, age, and presence of IgA class TG2 antibodies.

Age (Years)	Girls		Boys		All	
	n	%	n	%	n	%
11	238	16.3	251	17.2	489	33.5
12	243	16.7	253	17.3	496	34.0
13	233	16.0	240	16.5	473	32.4
11–13	714	49.0	744	51.0	1458	100
Presence of IgA class TG2-ab	1	0.14	1	0.13	2	0.14
Positive coeliac disease histology	1	0.14	1	0.13	2	0.14

## Data Availability

The data presented in this study are available within the article.
